# A high density recombination map of the pig reveals a correlation between sex-specific recombination and GC content

**DOI:** 10.1186/1471-2164-13-586

**Published:** 2012-11-15

**Authors:** Flavie Tortereau, Bertrand Servin, Laurent Frantz, Hendrik-Jan Megens, Denis Milan, Gary Rohrer, Ralph Wiedmann, Jonathan Beever, Alan L Archibald, Lawrence B Schook, Martien AM Groenen

**Affiliations:** 1 Wageningen University, Animal Breeding and Genomics Centre, PO Box 338, 6700AH, Wageningen, The Netherlands; 2 INRA, Laboratoire de Génétique Cellulaire, 31320, Castanet-Tolosan, France; 3 USDA, ARS, US Meat Animal Research Center, PO Box 166, Spur 18D, Clay Center, NE, 68933-0166, USA; 4 University of Illinois, Department of Animal Sciences and Institute for Genomic Biology, 382 ERML, 1201 W. Gregory Avenue, Urbana, IL, 61801, USA; 5 Division of Genetics and Genomics, The Roslin Institute and R(D)SVS, University of Edinburgh, Easter Bush, Midlothian Cedex, EH25 9RG, UK

**Keywords:** Pig, Recombination, Genome, SNP, Linkage, Meiosis, Telomere, Centromere, Isochore

## Abstract

**Background:**

The availability of a high-density SNP genotyping chip and a reference genome sequence of the pig (*Sus scrofa*) enabled the construction of a high-density linkage map. A high-density linkage map is an essential tool for further fine-mapping of quantitative trait loci (QTL) for a variety of traits in the pig and for a better understanding of mechanisms underlying genome evolution.

**Results:**

Four different pig pedigrees were genotyped using the Illumina PorcineSNP60 BeadChip. Recombination maps for the autosomes were computed for each individual pedigree using a common set of markers. The resulting genetic maps comprised 38,599 SNPs, including 928 SNPs not positioned on a chromosome in the current assembly of the pig genome (build 10.2). The total genetic length varied according to the pedigree, from 1797 to 2149 cM. Female maps were longer than male maps, with a notable exception for SSC1 where male maps are characterized by a higher recombination rate than females in the region between 91–250 Mb. The recombination rates varied among chromosomes and along individual chromosomes, regions with high recombination rates tending to cluster close to the chromosome ends, irrespective of the position of the centromere. Correlations between main sequence features and recombination rates were investigated and significant correlations were obtained for all the studied motifs. Regions characterized by high recombination rates were enriched for specific GC-rich sequence motifs as compared to low recombinant regions. These correlations were higher in females than in males, and females were found to be more recombinant than males at regions where the GC content was greater than 0.4.

**Conclusions:**

The analysis of the recombination rate along the pig genome highlighted that the regions exhibiting higher levels of recombination tend to cluster around the ends of the chromosomes irrespective of the location of the centromere. Major sex-differences in recombination were observed: females had a higher recombination rate within GC-rich regions and exhibited a stronger correlation between recombination rates and specific sequence features.

## Background

Linkage maps have been widely used to identify genomic regions that influence phenotypic traits. In addition to the expected advances in fine-mapping of Quantitative Trait Loci (QTL) [1,2], high-density linkage maps provide a framework for checking the assembly of genome sequences and for studies of the evolution of these genomes through the analysis of recombination. Indeed, recombination lies at the heart of every genetic analysis, and whereas linkage maps in the past were constructed primarily to aid in the generation of a physical map, linkage maps are currently being recognized as indispensable tools to study virtually every aspect of genome biology. Genomic features that have been shown to correlate with recombination rate include GC content, gene density, gene expression, epigenetic modifications, nucleosome formation, repetitive element composition, isochore structure, but also patterns of genetic variation and differentiation within and between populations. For this reason, increasingly dense recombination maps have been constructed in the so called ‘post-genomic era’ for species such as human and mouse, focussing on identifying hotspots of recombination, and, recently, variation in the use of these hotspots between populations and between sexes.

Despite the evident importance of accurate and comprehensive linkage maps in the post-genomic era, comprehensive maps are currently only available for a handful of vertebrate species (human, mouse, rat, cattle, dog, zebra finch and chicken). This limited coverage of the recombination landscape severely limits the possibility of drawing general conclusions about the recombination rates in genomes, particularly now that it is becoming increasingly clear that various mechanisms can work together in creating a very dynamic use of recombination hotspots over time [3-6].

In swine, the first linkage map covering all the autosomes plus the X chromosome of the pig was established in 1995 [7] and a denser map comprising about 1,200 markers was published in 1996 [8]. Two other linkage maps comprising around 240 loci were published in the late 1990s [9,10]. These four maps were mainly based on microsatellites, Restriction Fragment Length Polymorphisms (RFLPs) and protein polymorphisms. More recently, SNPs were added to these maps [11], but the resolution remained low with an average inter-SNP distance of 3.94 cM. With the advent of genome-wide high-density SNP chips, genetic maps can comprise an increasing number of markers. Until now, such high-density genetic maps, based on microsatellites and SNPs, have been computed for human [12], mouse [13], chicken [14,15], cattle [16] and dog [17]. With the release of Illumina's Porcine SNP60 BeadChip [18], it became possible to construct a high-density recombination map of the porcine genome. In this work, we present four recombination maps for four different pedigrees. A single set of SNPs was used, each SNP being informative in at least one of the four pedigrees. The recombination maps were estimated using *a priori* knowledge of the SNPs' order. This physical order of the SNPs was based on the position of the SNPs on the porcine Radiation Hybrid (RH) map [19] and on the positions of the SNPs in the pig genome sequence (build 10.2).

## Results

### Genotyping quality

The Illumina PorcineSNP60 BeadChip, which provides assays for 64,232 SNPs, was used to genotype the four studied pedigrees (ILL, UIUC, USDA, ROS; Table 1). The *a priori* order used to compute the recombination map comprised 44,760 SNPs: 35,098 from the RH order, and 9,662 derived from the sequence assembly. Of the 44,760 SNPs, 5,980 SNPs were discarded because of their low call-rate (<97%), and an set of 181 SNPs was removed because they exhibited a large number of Mendelian inconsistencies in several families. When Mendelian inconsistencies were only limited to one particular family per pedigree, genotypes were considered as missing in this family. A total of 168 individuals were removed from the four pedigrees because of their high proportion of incorrect genotypes due to either pedigree or genotyping errors. Finally, the average number of informative meiosis per marker was 432 for ILL, 200 for UIUC, 670 for USDA and 120 for ROS.

**Table 1:** Description of the four pedigrees

**Pedigree**	**Cross**	**F0 males**	**F0 females**	**F1 males**	**F1 females**	**F2**
**ILL**	F2	3 Berkshire	17 Duroc	5	44	595
**UIUC**	F2	3 Meishan	7 Yorkshire	3	15	260
**ROS**	F2 (reciprocal)	5 Meishan	6 Large White	2	16	151
		5 Large White	5 Meishan	4	14	
**USDA** *	overlapping F2	13	35	12	27	97
		13	55	10	53	376
		12	66	8	62	547

*The USDA pedigree was derived from a population composed of ¼ Duroc, ¼ Large White, ¼ maternal Landrace and ¼ high growth Landrace.

### Recombination maps

The *a priori* order, on which the recombination analyses were based, comprised 44,760 SNPs, including 556 SNPs mapped to unplaced scaffolds and 480 SNPs with no sequence match on the genome assembly. Finally, we were able to construct a genetic map with a total of 38,599 SNPs including 508 from unplaced scaffolds and 420 that had no match on the assembly. On average, there were 2,144 SNPs per chromosome, ranging from 1,011 (SSC18) to 5,293 (SSC1) (Table 2). This set of SNPs was chosen as being valid for all four pedigrees; recombination maps were calculated separately for each of them. The rates of phase reconstruction differed for the four pedigrees. For the complete genome, the highest rate was obtained for the UIUC pedigree (99.0%) and the lowest rate was obtained for the ROS pedigree (87.0%). The ILL and USDA pedigrees were intermediate with phase reconstruction rates of 96.5% and 92.0%, respectively.

**Table 2:** Description of the linkage maps of the four pedigrees

			**ILL**		**UIUC**		**USDA**		**ROS**	
**SSC**	**Nb SNP**	**Physical length (Mb)**	Linkage map (cM)*	cM/Mb^**^	**Linkage map (cM)**	**cM/Mb**	**Linkage map (cM)**	**cM/Mb**	**Linkage map (cM)**	**cM/Mb**
1	5293	308	145	0.37	144	0.38	130	0.33	140	0.37
2	2492	158	122	0.64	137	0.68	110	0.57	113	0.60
3	2044	141	120	0.74	122	0.76	113	0.71	106	0.65
4	2789	143	125	0.70	129	0.73	111	0.64	115	0.61
5	1737	109	114	0.83	124	0.94	97	0.73	104	0.89
6	2156	157	148	0.78	151	0.87	122	0.68	148	0.85
7	2693	132	132	0.89	144	0.97	117	0.78	119	0.78
8	2008	147	112	0.63	124	0.70	110	0.62	110	0.62
9	2166	153	127	0.74	135	0.81	117	0.69	112	0.63
10	1173	77	109	1.22	116	1.29	99	1.10	89	0.96
11	1332	86	85	0.77	96	0.89	77	0.70	73	0.62
12	1038	63	99	1.33	99	1.30	86	1.11	94	1.24
13	2875	216	113	0.45	122	0.51	97	0.40	117	0.47
14	3142	153	124	0.75	138	0.85	110	0.64	111	0.64
15	2085	154	108	0.61	123	0.67	97	0.54	110	0.61
16	1337	85	83	0.80	91	0.86	78	0.75	77	0.73
17	1227	68	78	0.94	83	1.05	67	0.81	76	0.98
18	1011	60	68	0.91	71	0.95	59	0.80	44	0.56
***TOTAL***	***38599***	***2334***	***2012***	***0.78***	***2149***	***0.85***	***1797***	***0.70***	***1858***	***0.71***

* Linkage map lengths are the sex-averaged map lengths, calculated by using all the individuals of the pedigree. **The ratio cM/Mb was calculated in bins of 1 Mb.

**Figure 1:**
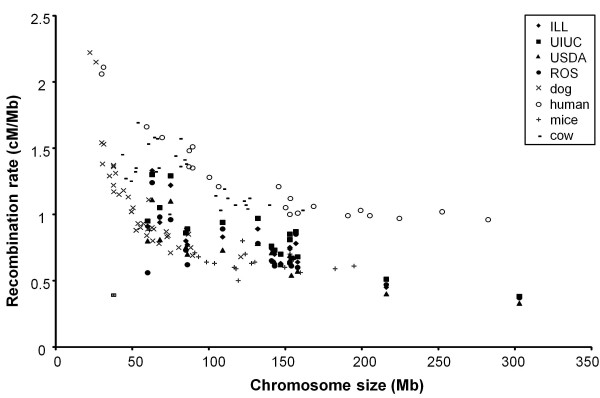
**Physical and genetic positions of the SNPs mapped on SSC1.** The ILL and UIUC positions are plotted as solid and dotted lines respectively, female maps being in black and male maps in grey.

The details of the genetic maps calculated for each of the four pedigrees are presented in Table 2. The estimates of the total genetic length of the 18 autosomes were 2,012 cM for ILL, 2,149 cM for UIUC, 1,797 cM for USDA and 1,858 cM for ROS. The largest chromosome was SSC6 for ILL, UIUC and ROS pedigrees with 148, 151 and 148 cM, respectively; whereas it was SSC1 for the USDA pedigree with 130 cM. SSC18 was the smallest chromosome for all the pedigrees, its length varying from 44 cM for the ROS pedigree to 71 cM for the UIUC pedigree. Estimates of the size of linkage maps are influenced by many factors. Recombination events are stochastic and different sub-sets of the markers (SNPs) are informative in the different pedigrees. Although potential genotyping errors were removed from the analysis, specific SNPs segregating only in particular pedigrees might still result in increased map length if they have a higher error rate. However, our observed difference in size between the ILL and UIUC maps versus the USDA and ROS maps, is consistently seen for most of the chromosomes, indicating a true biological difference in the recombination rate for these different crosses. Because within the USDA and ROS pedigrees female recombination was not well taken into account (due to the low number of offspring per dam or because of missing genotypes), male and female recombination maps were described separately only for the ILL and UIUC pedigrees (Table 3). Consistent with findings in other mammals, the total lengths were longer for the female maps (2,244 and 2,545 cM for ILL and UIUC respectively) than for the male maps (1,782 and 1,747 cM for ILL and UIUC respectively). SSC1 stands out as an exception, with the male maps being longer than the female maps. This difference is due to a low recombination rate in the females in the region between 90 and 250 Mb (Figure 1). In this 90–250 Mb region, the average recombination rate in females was 0.056 and 0.031 cM/Mb for ILL and UIUC respectively whereas it was 0.286 and 0.290 for males in ILL and UIUC pedigrees respectively.

**Table 3:** Description of sex-specific linkage maps of the ILL and UIUC pedigrees

	**ILL *♀***		**ILL *♂***		**UIUC *♀***		**UIUC *♂***	
**SSC**	**Linkage map (cM)**	cM/Mb^*^	**Linkage map (cM)**	**cM/Mb**	**Linkage map (cM)**	**cM/Mb**	**Linkage map (cM)**	**cM/Mb**
1	138	0.34	151	0.40	129	0.33	159	0.42
2	133	0.70	111	0.58	167	0.83	107	0.53
3	130	0.80	111	0.69	135	0.85	109	0.67
4	137	0.76	112	0.63	150	0.87	108	0.60
5	128	0.93	100	0.74	147	1.05	100	0.82
6	170	0.89	125	0.68	181	1.05	120	0.69
7	146	0.96	118	0.82	176	1.17	112	0.77
8	120	0.66	105	0.60	140	0.78	108	0.63
9	144	0.85	110	0.64	167	1.00	103	0.63
10	126	1.41	93	1.03	140	1.53	91	1.05
11	107	0.95	63	0.59	118	1.07	74	0.72
12	117	1.58	81	1.08	124	1.70	74	0.90
13	115	0.46	112	0.44	133	0.51	110	0.44
14	135	0.84	112	1.01	163	0.66	112	0.69
15	115	0.63	102	0.58	151	0.81	95	0.53
16	96	0.90	70	0.70	119	1.13	62	0.59
17	100	1.19	56	0.70	114	1.40	52	0.70
18	87	1.17	50	0.65	91	1.23	51	0.68
**TOTAL**	**2244**	**0.89**	**1782**	**0.70**	**2545**	**1.00**	**1747**	**0.67**

* The ratio cM/Mb was calculated in bins of 1 Mb.

### Recombination rates

**Figure 2:**
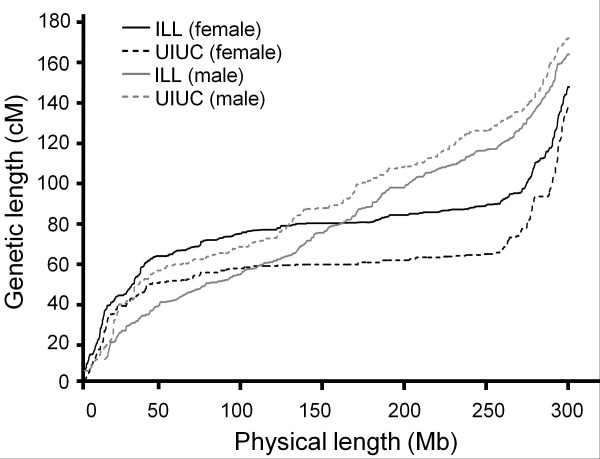
**Distribution of the recombination rate according to the physical chromosome size.** Results are given for the four pig pedigrees in black (squares for ILL, diamond for UIUC, triangles for USDA and circles for ROS), and for other mammals in grey (cross for dog, circle for human, plus for mice and dash for cattle).

Recombination rates were calculated for non-overlapping bins of 1 Mb with marker positions delimiting the intervals (Additional file 1). At the level of the genome, the highest average recombination rate was obtained for the UIUC pedigree with 0.85 cM/Mb, the lowest being obtained for the USDA pedigree with 0.70 cM/Mb (Table 2). This ratio was highly variable depending on the physical length of the chromosomes, the shortest ones having higher ratios than the longest ones (Figure 2).

**Figure 3:**
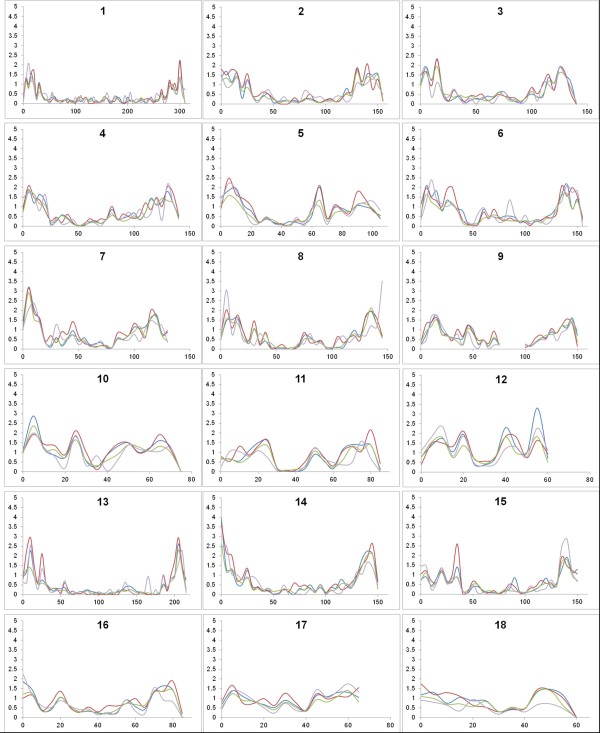
**Recombination rate for the four pedigrees.** Recombination rates were calculated for bins of 1 Mb and plotted using a moving average of 5 Mb. On the x-axis, the genomic position is given in million base pairs. On the y-axis, the recombination rate is given in cM/Mb. Results for ILL, UIUC, USDA and ROS pedigrees are given in blue, red, green and grey respectively.

For the four pedigrees, the highest recombination rate was observed for SSC12 with values of 1.33, 1.30, 1.11 and 1.24 cM/Mb for ILL, UIUC, USDA and ROS, respectively. The lowest recombination rate was obtained on SSC1 with 0.37, 0.38, 0.33 and 0.37 cM/Mb for ILL, UIUC, USDA and ROS respectively (Table 2). At the genome level, recombination rates were higher in females than in males. At the chromosome levels, only SSC1 displayed higher recombination rates in males than in females, for ILL and UIUC pedigrees (Table 3). The distribution of recombination rates was not constant along the chromosomes with high recombination rates mostly concentrated around the end of the chromosomes (Figure 1 and Figure 3). This is seen both in male and female recombination but the effect is somewhat stronger in female recombination. Overall, the recombination maps for the 4 pedigrees are in good agreement, although small local differences can be detected.

**Figure 4:**
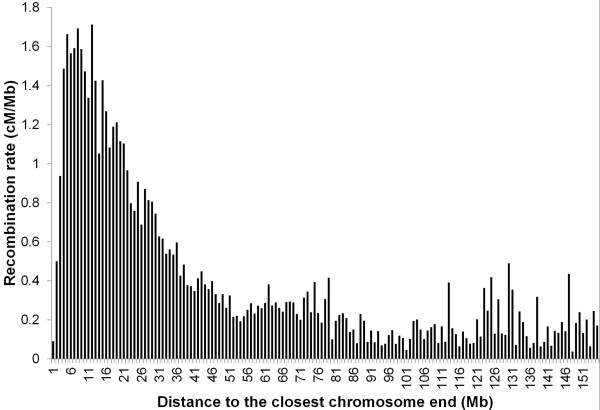
**Pig recombination rate distribution according to the distance (in Mb) to the closest chromosome end.** Recombination rate (cM / Mb), GC content.

On SSC9, the large gap observed is due to the absence of SNPs that could be reliably included for the four pedigrees in the genetic maps. The distribution of the recombination rates plotted against the physical distance to the closest chromosome end confirm that high recombination rates tend to cluster around the chromosome ends, irrespective of the position of the centromere (Figure 4). For the sex-averaged map, the correlation between the recombination rate and the physical distance to the closest chromosome end was estimated to be-0.48 (p-value < 0.0001), and correlations for separate male and females maps were identical.

### Correlation of recombination with sequence parameters

Correlations between recombination rates and various sequence parameters (GC content, repetitive elements content and short sequences) have previously been observed in human [12], chicken [14], dog [17] and mouse [13]. The occurrence of these sequence parameters was calculated within bins of 1 Mb and the correlations with the recombination rates were estimated. With the sex-average map, all sequence features were highly significantly correlated with the recombination rate (p-value <0.05). However, the level of the correlations was lower for LINEs and LTRs, with Pearson correlation coefficients of-0.05 and 0.06, respectively. The comparison of the sequence composition of recombination ‘jungles’ and ‘deserts’ (1 Mb intervals with the 10% highest and 10% lowest recombination rates respectively) also highlights this link between the occurrence of specific sequence features and recombination rate (Table 4). Recombination jungles were enriched in specific GC rich motifs as compared to the deserts. The largest difference was observed for the CCCCACCCC sequence, this sequence being almost three times more frequent in recombination jungles than in deserts.

**Table 4:** Correlations between recombination rate and sequence composition in 1 Mb bins

	**Sex-average**					**Male**					**Female**				
**Motif**	**Correlation**	**P-value**	**J**	**D**	**J/D**	**Correlation**	**P-value**	**J**	**D**	**J/D**	**Correlation**	**P-value**	**J**	**D**	**J/D**
GC%	0.34	<0.0001	0.45	0.38	1.19	0.15	<0.0001	0.43	0.40	1.09	0.44	<0.0001	0.46	0.38	1.21
Line	−0.05	0.0227	458	492	0.93	0.03	0.1643	477	485	0.98	−0.08	<0.0001	456	494	0.92
Low-complexity	−0.26	<0.0001	131	174	0.75	−0.15	<0.0001	139	165	0.84	−0.31	<0.0001	129	173	0.75
LTR	0.06	0.0037	136	132	1.03	0.07	0.0011	136	133	1.02	−0.01	0.5137	131	135	0.97
Simple repeat	0.31	<0.001	179	150	1.19	0.18	<0.0001	174	158	1.10	0.31	<0.0001	179	150	1.19
SINE	0.35	<0.0001	754	506	1.49	0.18	<0.0001	690	587	1.18	0.43	<0.0001	785	492	1.60
CCTCCT	0.34	<0.0001	463	294	1.57	0.17	<0.0001	424	339	1.25	0.41	<0.0001	474	291	1.63
CCTCCCT	0.37	<0.0001	159	82	1.95	0.19	<0.0001	143	103	1.38	0.45	<0.0001	166	81	2.05
CTCTCCC	0.38	<0.0001	137	79	1.72	0.21	<0.0001	125	93	1.34	0.44	<0.0001	141	80	1.76
CCCCCCC	0.41	<0.0001	184	64	2.88	0.21	<0.0001	155	89	1.73	0.49	<0.0001	193	62	3.10
CCCCACCCC	0.36	<0.0001	52	18	2.91	0.17	<0.0001	43	28	1.52	0.45	<0.0001	56	17	3.30
CCNCCNGGNGG	0.25	<0.0001	24	8	2.84	0.09	<0.0001	19	12	1.52	0.33	<0.0001	25	7	3.41
CCNCCNTNNCCNC	0.36	<0.0001	44	17	2.68	0.19	<0.0001	37	24	1.55	0.44	<0.0001	47	16	2.95

Columns J and D represent the average composition (GC content and counts of motifs) of Jungle and Desert regions respectively. The J/D ratio represents the comparison of these compositions, a value higher than one indicating that the motif is more frequent in Jungle than in Desert regions.

**Figure 5:**
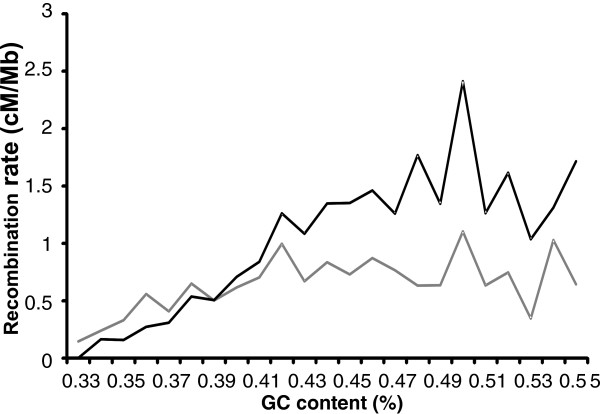
**Distribution of recombination rate within males and females in relation to the GC content.** Black and grey bars represent female and male recombination rates respectively.

Male and female recombination rates were also analysed separately and large differences were observed. The correlation of the recombination rate with GC content was higher in females (0.44) than in males (0.15) (Table 4). In agreement with this is the observation that in females recombination is higher only when the GC content of the region is higher than 0.40 whereas it is lower for regions where the GC ratio is smaller than 0.39 (Figure 5).

Jungle/desert ratios were also highly different between sexes for SINEs and short sequence motifs. In females, this ratio reached 3.41 for the CTCF consensus sequence (CCNCCNGGNGG), whereas it only reached 1.52 in males.

## Discussion

### Genetic maps

The reliability of a recombination map is of major importance for linkage and genome-wide association analyses [1]. The presented recombination maps were computed for four different pedigrees, with a subset of SNPs being optimal for all of them, finally comprising 38,599 SNPs. Because only SNPs for which sequence and RH positions were in agreement were included in the analyses and because the recombination maps confirmed the *a priori* order, the map presented in this study is expected to be as accurate as possible with currently available data. The map presented in this paper is the densest recombination map ever computed for the porcine genome. Until now, the shortest average marker interval on a genetic map was reached by the USDA MARC map [8] with an average interval of 2.23 cM. The large number of SNPs as well as the high number of informative meiosis included in the present analysis enabled the computation of a high-density recombination map of the porcine genome with a consequent substantial increase in resolution (around 0.1 cM) compared to previous maps. The total length of the genetic map varied between the four pedigrees, from 1,797 cM to 2,149 cM, which is smaller than the previously published genetic maps. This decrease in the total length of the map can in part be explained by the lower rate of genotyping errors with SNP chip genotyping as compared to microsatellites or RFLP genotyping. Another factor that contributes to the decreased map size is the fact that male meioses contributed most to the current map, while the USDA maps [8,20] were based primarily on female meioses. Concerning the map computed with gene-associated SNPs [11], the sex-averaged genetic maps presented in our study are 15 to 45% shorter, if we take into account only the regions covered in both studies [11]. The same is observed for the sex-specific maps. Female maps are 21 to 33% shorter in our study, and the two male genetic maps are around 18-19% shorter than the one presented by Vingborg *et al.*[11]. Recently, two genetic maps based on the 60 k SNP chip have been published for Landrace and Duroc, with similar chromosome lengths as in our study except for SSC1 where a length of 199.8 cM was obtained in Landrace, very different from all the others [21].

### Recombination rates

The recombination map of the porcine genome described in this paper, revealed major chromosomal as well as regional differences in recombination rates. The four pedigrees clustered into two different groups, ILL and UIUC having recombination rates close to 0.8 cM/Mb whereas the two other pedigrees had lower recombination rates close to 0.7 cM/Mb. All these values are in the range of previous findings in mammals (from 0.6 cM/Mb in mouse [13] to 1.25 cM/Mb in cattle [16]). In birds, the observed recombination rate is higher with a value of 1.5 cM/Mb in the zebra finch [22] and up to 2.7 to 3.4 cM/Mb in chicken [15]. Differences in recombination rate within a species have already been described in mice [23] and chicken [14,15]. Differences in recombination rate observed in this study among the four pedigrees are partly explained by the percentage of phases that could be reconstructed. A lower number of phases could be reconstructed in the two pedigrees in which family sizes were small (USDA) or where several mother genotypes were missing (ROS). Another potential cause for the observed differences are sequence variations within the individuals used, and in particular structural variants like copy number variants and local inversions. In particular the UIUC and ROS crosses involving Chinese (Meishan) and European (Large White/Yorkshire) breeds which diverged around 1 million years ago [24], are likely to have local inversions that would affect recombination at these positions.

In addition to these differences among the four pedigrees studied, the recombination rate also varied among chromosomes (Table 2 and Figure 2) as well as within chromosomes (Figure 1). The distribution of the recombination rate according to the physical size of the chromosomes obtained with the pig was in agreement with the distributions observed in other mammalian species and birds: shortest chromosomes exhibiting higher recombination rates. This result is in line with the observation of at least one cross-over occurring per meiosis per chromosome [25]. It is noteworthy that for the longest chromosomes in pig, the overall recombination fraction (cM/Mbp) is much lower than for any other mammalian species for which recombination maps have been developed to date (Figure 2).

The distribution of the recombination rate according to the distance to the closest chromosome end showed that higher recombination rates were mostly observed towards the ends of the pig chromosomes. Moreover, the position of the centromere did not seem to influence this distribution: e.g., SSC13 is an acrocentric chromosome and the distribution of the recombination rate along this chromosome is very similar to the distribution along metacentric or submetacentric chromosomes (pig chromosomes 1 to 12 being meta- or submetacentric chromosomes, the others being acrocentric chromosomes [26]). Other species with acrocentric chromosomes, such as the dog, show a marked increase in recombination fraction at the medial and centromeric parts of most chromosomes [17]. The general absence of this pattern in the acrocentric chromosomes in pigs raises questions on how and particularly when the pig chromosomes became acrocentric. The evolution of centromere positions can be highly dynamic, and the current apparent disparity between centromere position and recombination rate may hint at a recent shift of the position of the centromere in several pig chromosomes.

In human and rat, recombination rates were also found higher in the telomeric regions and reduced close to the centre of the chromosomes [27], but this pattern is not as pronounced as in the pig. This preferential distribution of crossing overs at the chromosomal ends is even more striking in zebra finch with long central regions where the recombination rate remains extremely low [22]. However, in the zebra finch, and also in chicken, these telomeric regions of exceptionally high recombination compared to the other parts of the chromosomes seem to be much more confined to the extreme edges of the chromosomes, whereas in the pig these distal regions of high recombination are less pronounced but much greater in size. In some species, however, this particular distribution of recombination rate along a chromosome is not observed. In the mouse, the correlation estimated between recombination rate and the distance to the centre of the chromosome does not differ from the one estimated with respect to the distance to the telomere [27], which is in agreement with the distribution of the recombination rate estimated from the sex-averaged genetic map [13]. Similarly, the plot of the genetic map against the physical map of the bovine genome does not show this sigmoid-like pattern that indicates higher recombination rates at the chromosome ends [16]. What is particularly striking in the pig, is that this elevated recombination towards the ends of the chromosomes is also seen for the acrocentric chromosomes. Previous observations in other mammals, were interpreted as that recombination at centromeric regions was low, because recombination would interfere with kinetochore assembly [28] at the centromers. Unless the pig has evolved specific features to overcome such interference, which does not seem to be very likely, other yet unknown structures of mammalian chromosomes underlie these observed differences.

### Recombination and sequence features

In this study, we show that recombination rates vary with the distance to the closest chromosome end. In human, the GC content was negatively correlated with the distance to the chromosome end [29], and the porcine genome exhibits the same negative correlation. The GC content has also been shown to be strongly positively correlated with recombination rates in human [12,30,31], mice [13], chicken [14] and zebra finch [22], and this was also confirmed in this study. This seemingly universal positive correlation between GC content and recombination is thought to signify a shared underlying mechanism determining recombination rates [32,33], although it has been proposed that higher GC content can conversely be the result of high recombination rate [34,35].

Mechanisms explaining the direct relationship between GC content and recombination rate identify the presence of certain recognition motifs for DNA binding proteins that have a known function in meiosis or the recombination process directly, such as cohesin and PR domain-containing protein 9. In other mammalian and avian species, high-density linkage maps have shown strong correlations between recombination rates and various sequences such as the consensus cohesion binding site; the 7-nucleotide oligomer CCTCCCT [4,13] and a 13-nucleotide oligomer described in human CCNCCNTNNCCNC [3]. Recently, it was shown that this 13-nucleotide sequence is recognized in vitro by the human PR domain-containing protein 9, encoded by the *PRDM9* gene [4]. The PR domain-containing protein 9 is known to regulate recombination hotspot activity in human [5]. GC-rich motifs have been investigated in this study and all of them are overrepresented in recombination jungles and underrepresented in deserts. The sequences CCTCCCT and CCCCACCCC, overrepresented in about 10% of human hotspots [3] are also correlated with higher recombination rates in mouse and chicken, jungle/desert ratios being close to 2 or higher. The same is observed in this study with a ratio close to 2 or higher (Table 4).

### Sex-differences

In our study, male and female maps were analysed separately for the ILL and UIUC pedigrees. In both designs, female meioses were better sampled than in the two other pedigrees for which dams were not always genotyped or had too few offspring. The ROS and USDA maps are thus closer to male maps that can be explained by their shorter lengths as compared to the sex-average maps of ILL and UIUC. It should also be noted that the length of the female maps that are reported here are close to the original MARC map that was based primarily on female meioses [8].

In most species, the heterogametic sex is expected to have a lower recombination rate than the homogametic sex [36]. This was confirmed in this study at the level of the genome with female maps being longer than male maps by 26% or 46% for ILL and UIUC pedigrees, respectively. However, SSC1 stood out with more recombination events described within males than within females. As shown in Figure 1, females displayed a 160 Mb region with a very low recombination frequency. Vingborg et *al.*[11] found that SSC1 was longer in females than in males, but the 70–100 cM region of SSC1 also displayed higher recombination in males than in females [11]. The greater genetic length of SSC1 in males as compared to females was already observed in previous pig genetic maps [7,37-39]. All these previous maps were based on crosses between genetically diverse founder/grandparental animals including Wild Boars and European commercial breeds [37] and Chinese and European breeds [8,39] or combinations thereof [37]. The current study also included highly diverse pedigree origins, which makes breed effects therefore unlikely to be the major explanation for this locally low recombination rate. For the ILL pedigree, we observed a small difference between the male and female maps of SSC13 and this was also reported by Guo *et al.*[39] who observed a female to male ratio of 0.98 for this chromosome. In the linkage map computed with gene-associated SNPs, SSC13 was also found to be rather similar in males and females [11]. For this chromosome, we did not observe such large sex-differences in the distribution of the recombination rates along the chromosome as for SSC1. To better understand this apparent discrepancy in recombination rates between male and female on different chromosomes, we plotted the recombination rates as a function of GC content for male and female separately (Figure 5). Although in both sexes higher average recombination frequencies were observed for regions exhibiting a higher GC content, this correlation was much greater in females than in males. This also explains why, contrary to what is observed in most other mammals [6], there is a tendency of females to show even more elevated recombination towards the ends of the chromosomes than the males. In fact, males showed a clear lower recombination rate at AT rich regions, but females showed an even lower recombination at AT rich regions relative to males. This resulted in an overall lower recombination rate in females in AT rich regions than observed in males. This may explain the observation on SSC1, where the recombination was higher in males due to the 90–250 Mb region being relatively AT rich (GC content of 0.39 compared to the genome average of 0.42). This effect was only clearly observed on SSC1 since the other chromosomes lack such long regions of low GC content. A positive correlation between recombination rates in female and GC content had already been reported in human [40], and this was confirmed in the present analysis (Table 4). Recombination in males appeared to be less sensitive to the frequency of the GC rich motifs and the observed jungle / desert ratios are much higher in females.

The positive relationship between GC content and female recombination does not appear to be universal. Sex-specific GC related recombination rates for instance have been observed in dogs, but appears to be opposite in this species: higher GC content appears to be negatively correlated with female recombination rate [17]. Since the study on dog recombination did not dissect the precise relationship of male and female recombination rates as a function of GC content as done in the present study it is difficult to compare the results. However, this opposite relationship in dogs may hint at specific recombination mechanisms that apply to acrocentric vs. metacentric karyotypes, and demonstrates the importance of having detailed recombination maps for many different species for comparative genome biology purposes.

Even if the mechanisms underlying sex differences in recombination are largely unknown, a number of mechanisms for sex-specific differences have been proposed: difference in time allotted for so called bouquet formation in meiosis [6], difference in the compactness of the chromosomes at pachytene phase of meiosis [41], genomic imprinting [6], or differences in the use of specific recombination-hotspot specific motifs [12,41]. For instance, it has been shown that different alleles of the *RNF212* gene can have opposite effects on male and female recombination rate [12]. In mice, a QTL analysis was carried out to detect regions of the genome underlying recombination rate and the most significant QTLs were observed on chromosome X [42]. This raises the possibility that chromosomes X and/or Y may be involved in the observed striking difference of recombination rates between males and females. However, the analysis included only males, so no sex-specific QTL could be analysed. This study in mice indicated that genomic variations on the X chromosome influenced the recombination rate, but it did not provide further explanation of why females recombine more than males. Finally, in mice, the analysis of meiocytes from XX females, XY males, XY sex-reversed and XO females indicated that recombination patterns depend more on being a male or a female than on the true chromosomal genotype [43]. All of these mechanisms may be compatible with the patterns observed in the present paper. In fact, the evolution of recombination and recombination hotspots seems highly dynamic, and may involve universal (e.g. chromosome compactness at the pachytene phase at meiosis) and species specific mechanisms (e.g. use of sex specific hotspots). The importance of each of these mechanisms will need to be tested for various species using higher density linkage maps in the future.

## Conclusions

In this study we present the first high-density recombination map of the porcine genome, with a resolution substantially higher than previously published maps. This high resolution enabled us to focus on the differences between low and high- recombining regions of the genome, and on the large differences that we observed between males and females. As expected, at the genome level, female maps were longer than male maps. The unexpected higher recombination rates in males observed on SSC1, could be explained by a large region of low GC content where females showed very low recombination rates. The higher correlation between recombination rate and GC content (as well as GC rich motifs) in females as compared to males was confirmed at the genome level. Until now, this high correlation between recombination rates in females and GC content has only been reported in human. Further analyses of the mechanisms underlying recombination are needed to identify the molecular mechanism underlying this observed difference. The increased insight into the porcine recombination landscape will help future studies aimed at understanding the evolution of the pig genome and at fine-mapping identified QTLs for economically important traits.

## Methods

### Mapping populations and SNP genotyping

The animals used to compute the recombination maps belong to four independent pedigrees. Three were based on an F2 design (including one reciprocal cross) and one was based on multi-stage crosses. Details about the four pedigrees are presented in Table 1.

To compute recombination maps, only families with more than four full-sibs were retained in the analysis. Therefore, recombination maps were calculated based on the information from 573 animals of the ILL pedigree, 247 from the UIUC pedigree, 204 from the ROS pedigree and 1298 from the USDA pedigree. The four pig pedigrees were genotyped using the Illumina PorcineSNP60 BeadChip (San Diego, CA, USA). Each pedigree was genotyped independently, and a total of 664 samples from ILL, 337 from UIUC, 208 from ROS and 1337 from USDA were genotyped. To carry out the computation of recombination maps, only SNPs with a call rate higher than 97% were retained. In addition, all the genotypes were checked for Mendelian inheritance and erroneous genotypes were set as missing. Double recombinants at specific markers were considered as genotyping errors and the corresponding genotypes were therefore set as missing.

### Recombination map calculation

Recombination maps were computed for each pedigree independently using a single set of SNPs, each SNP being informative in at least one of the four pedigrees. The first step of the recombination map calculation was to determine the best physical order of the markers based on the RH mapping [19] and in silico mapping of the SNPs to the pig genome sequence. The genotyping of the two RH panels of the porcine genome on the PorcineSNP60 BeadChip enabled the computation of a physical map [19]. SNPs were positioned on the current pig genome sequence build 10.2 ( ftp://ftp.ncbi.nih.gov/genbank/genomes/Eukaryotes/vertebrates_mammals/Sus_scrofa/Sscrofa10.2/) by aligning the 200 bp sequence adjacent to the SNP against build 10.2 using BLAT [44]. The RH order was considered as the basic order and when it was consistent with the sequence assembly, SNPs from the assembly were included in the best physical order.

The second step was the estimation of the recombination rates along chromosomes using the method described by Coop et al. [45]. Briefly, haplotypes transmitted by a parent to each of its offspring were inferred based on informative SNPs. Then, within a given nuclear family, one of the offspring (template) was successively compared to the others: at a marker, it was deduced whether both offspring were Identical By Descent (IBD) or not. Any switch from an IBD to a non-IBD status indicated a recombination event. Regions where the majority of offspring showed a recombination were considered as indicative of a recombination in the template offspring. Finally, the parental phases were partially reconstructed, allowing identification of recombination events that occurred in each meiosis [45]. Recombination rates were transformed into centimorgans (cM) using the Haldane mapping function.

As a result, four recombination maps were computed and recombination rates in cM/Mb were calculated for each pedigree along the genome. These recombination rates were estimated in non-overlapping bins of approximately 1 Mb considering the exact SNP positions as the delimiters of the bins. An average recombination rate was also estimated along the genome over the four pedigrees and was used to carry out further analyses in relation to correlation with sequence features. Similarly, female and male recombination rates were estimated along the genome.

### Correlation of recombination with sequence parameters

The average recombination rate was compared to the distribution of various sequence motifs including repetitive elements (LINEs, SINEs, LTRs, simple repeats and low-complexity repeats), GC content, and GC rich motifs previously shown to be correlated with high recombination rates (CCTCCT, CCTCCCT, CTCTCCC, CCCCCCC, CCCCACCCC, the CTCF consensus sequence CCNCCNGGNGG and the PRDM9 consensus binding sequence CCNCCNTNNCCNC). The distribution of sequence motifs and GC content were calculated for bins of 1 Mb using the current assembly (build 10.2) and the correlations with recombination rates were tested using Pearson's correlation coefficient with the CORR procedure in SAS (SAS® 9.1, SAS Institute, Inc.). Similar results were obtained using the more conservative Spearman test (data not shown). To further investigate the link between sequence features and recombination rate, the sequence composition of jungle and desert regions [13] were compared. Jungle regions were defined as the 1 Mb intervals with the 10% highest recombination rates, and conversely, desert regions were defined as the 1 Mb intervals with the 10% lowest recombination rates. The sequence composition of these Jungle and Desert regions were compared to detect whether there is a particular enrichment in some motifs in one of the two regions. A J/D ratio higher than one, indicates that the motif is more frequent in regions with high recombination rates than in regions with low recombination rates. Conversely a ratio lower than one indicates that the motif is more frequent in regions with low recombination rates. These ratios were also estimated independently in males and females. Finally, the correlation between recombination rate and the physical distance to the closest chromosome end was also estimated.

## Abbreviations

IBD: Identical By Descend; QTL: Quantitative Trait Loci; RFLP: Restriction Fragment Length Polymorphism; RH: Radiation Hybrid; SNP: Single Nucleotide Polymorphism.

## Competing interests

The authors declare that they have no competing interests.

## Authors' contribution

FT calculated recombination frequencies and wrote the paper; BT developed the program to calculate the recombination frequencies and RH mapping of the SNPs; LF analysed correlations with sequence features and GC percentage; HJM was involved in the analysis and discussion of the correlation between the genomic landscape and recombination frequency; DM performed RH mapping of SNPs; RW and GR SNP genotypes of the USDA pedigree; JB SNP genotypes of the ILL pedigree; ALA SNP genotypes of the ROS pedigree; LBS genotypes of the UIUC pedigree; MAMG overall coordination and finalizing the paper. All authors were involved in improving the manuscript. The final manuscript version was reviewed and approved by all the authors.

## Supplementary Material

Additional file 1 Average recombination rates (based on all 4 pedigrees) for all 1 Mb bins. Click here for file
